# Local Analysis of Human Cortex in MRI Brain Volume

**DOI:** 10.1155/2014/983871

**Published:** 2014-01-29

**Authors:** Sami Bourouis

**Affiliations:** ^1^Université de Tunis El Manar, École Nationale d'Ingénieurs de Tunis, BP 37 Belvedere Tunis, 1002 Tunis, Tunisia; ^2^College of Computers and Information Technology, Taif University, P.O. Box 888, Taif 21974, Saudi Arabia

## Abstract

This paper describes a method for subcortical identification and labeling of 3D medical MRI images. Indeed, the ability to identify similarities between the most characteristic subcortical structures such as sulci and gyri is helpful for human brain mapping studies in general and medical diagnosis in particular. However, these structures vary greatly from one individual to another because they have different geometric properties. For this purpose, we have developed an efficient tool that allows a user to start with brain imaging, to segment the border gray/white matter, to simplify the obtained cortex surface, and to describe this shape locally in order to identify homogeneous features. In this paper, a segmentation procedure using geometric curvature properties that provide an efficient discrimination for local shape is implemented on the brain cortical surface. Experimental results demonstrate the effectiveness and the validity of our approach.

## 1. Introduction

### 1.1. Problem Statement

**Figure 1:**
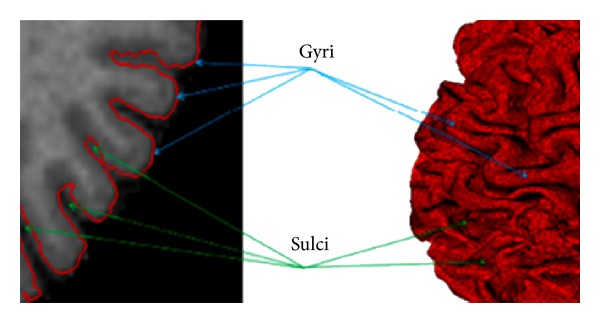
Illustration of sulcal and gyral patterns.

In a medical context, cortical surfaces analysis has motivated many researchers in anatomy, visualization, image registration, and pattern recognition [1–5]. The interest in this analysis comes in part from the variability of the convoluted shape of the cerebral cortex which is considered as the key of human intelligence and one of the most important factors regarding the comparison of brain anatomy and function [3]. However, the cortical surface has a complex structure comprised of folds “gyri” and fissures “sulci” (Figure 1). The accurate identification and labeling of these structures is helpful in human brain mapping studies, yet very challenging. Sulcal and gyral features are often considered as anatomical landmarks for automatically parcellating the cortex into features of interest that are functionally distinct. They also vary greatly from one individual to another because they have different geometric properties. So, we can consider them as a useful benchmark for comparison in the case of morphometric study [5]. For all these reasons, an accurate analysis of the cortical surface is very helpful for specialists in order to clarify the evolution differences between humans and animals, to study nervous disorders, and to preserve the main functions of a patient during brain surgery.

The labeling of cortical surface would be a significant aid in the study of the cortex anatomy. Indeed, the neurosurgeon would need to locate the regions of interest versus cortical folds (sulci and gyri) of the patient. However, this operation is not easy due to the complexity of anatomical shapes and the interpatient variability. Therefore, the extraction and labeling of cortical surface automatically and objectively become of great importance. It should be noted that this segmentation addresses various problems such as 3D visualization, topological analysis of the surface, brain mapping, and the analysis and the interpretation of brain activity. In addition, we think that automatic extraction can improve the computational time, the quality, and the reproducibility of this process.

### 1.2. Related Work

In this context, several studies have been developed to quantify cortical morphometric dissimilarities and then to facilitate the labeling of the human cortex. Unfortunately, some of them require manual intervention which penalizes the reproducibility of this task. Proposed approaches in the literature are divided into two main categories. The first one is based on spatial normalization. In this case, a coordinate system (i.e., 3D Talairach grid) is used to make a comparison between the patient brain and the reference (template) one. Template brain serves to give precise information on the point location. For example, Jaume et al. [6] proposed an algorithm to match an atlas labeled mesh to the patient brain mesh in a multiresolution way. Then, they transfer the labels from the matched mesh to label the patient mesh without manual intervention. Labeling the patient brain surface provides a map of the brain folds where the neurosurgeon can easily track the features of interest. The second category is based on the extraction of relevant anatomical regions (i.e., sulci, subcortical nuclei, gyri) in order to make comparison with shape descriptors or any other kind of descriptor. The manual delineation of these features requires a tedious process because of the large inter- and intraindividual variability [7]. So, the development of automatic segmentation tools based on specific and reproducible features is highly recommended [8–10]. The main features used for segmenting the cortical surface are based on geometric properties such as curvature [11–14], geodesic depth [15–17], medial axis [18], and so forth. For example, curvature-based approaches make the first-order approximation that sulci are concave and gyri are convex, and geodesic depth-based approaches use ad hoc techniques to distinguish these two complex structures. Kao et al. [17] apply depth thresholding to extract sulcal regions. They compute a geometric depth measure for each point on the cortical surface, and they extract sulcal regions by checking the connectivity above a depth threshold. Finally, they delineate the fundus by thinning each connected region keeping the endpoints fixed.

Other segmentation methods use other techniques such as watersheds of curvature function [19, 20]. Nevertheless, the watershed method shows often sensitivity to the choice of the depth threshold parameter and to noise, so, the division of the surface into regions cannot give accurate information about the differences between local regions. Moreover, deformable models [21] are applied to discriminate sulci and gyri. However, parameters describing the elasticity of the model affect the definition of some areas such as sulcal ones. The proposed approach in [14] is intended to describe a surface by dividing it into homogeneous regions according to the discrete mean and Gaussian curvature estimates. The surfaces are obtained from three-dimensional imaging datasets by isosurface extraction after data presmoothing. A hierarchical multiresolution representation of the isosurface is then generated. Finally, segmentation is performed at various levels of detail to detect the main features of the surface. This low-resolution description is used to determine constraints for the segmentation at the higher resolutions. Methods of extracting the cortical surfaces from MRI brain volumes have facilitated studies on inter- and intraindividual variability of sulcal and gyral patterns. In a clinical routine, these patterns are often delineated by a manual labeling process. This process is extremely tedious, time consuming, and probably leads to measurement errors.

### 1.3. Motivations and Contributions

According to the literature reviewed, we noted that the local curvature provides an effective shape measure and could be applied to discrete surface analysis. Moreover, measures related to the curvatures are used in several works to segment subcortical features. For example, the average principal curvatures (mean curvature) can describe the local folding of the surface. The gyri correspond to large values of the mean curvature and are detected via thresholding. Moreover, the Gaussian curvature is an intrinsic measure typically used to classify the surfaces in different primitive forms. Now, if we proceed by segmenting the cortex at high resolution, it would be difficult to interpret and compare the results because fine structures like gyri and sulci are very complex. To resolve this problem, we have to keep only main folds while eliminating the details. So, it is important to create a low-resolution representation of the initial mesh. This need is due also to rendering speed reasons and to allow fast transmission of 3D models in network-based applications.

This work takes place in this growing area and it proposes an efficient method and tool to segment and describe locally the subcortical structures. Unlike our previous work [22], the proposed work herein is more relevant and complete. Indeed, we treat in this paper the problem of local segmentation of human cerebral cortex. Our main purpose is to analyze finely obtained discrete surfaces after a segmentation operation of MRI volume. So, we propose here a fully automatic method for parcellating the cerebral cortex into gyri and sulci features of interest. It is based mainly on discrete differential geometry operators, which are the key to distinguish the two patterns, and on multiresolution representation to simplify and accelerate processing time.

The rest of this paper is organized as follows. The suggested method is presented in the next section. The experimental results are given in Section 3. Finally, Section 4 concludes the paper.

## 2. Methods and Materials

### 2.1. Method Outline

The proposed method is based mainly on two essential steps: the first one is intended to simplify the initial mesh and the second one to classify the surface as containing homogeneous attributes by using invariant local descriptors based on local geometric curvature. Because the cortex is composed of a large set of folds, it is important to detect only the main folds and not all small ones on the cortical surface. Progressive mesh simplification solves this problem. Indeed, the segmentation in high resolution could fail to properly distinguish between the major folds and it could lead to misinterpretation. Mesh simplification, whose role is to keep only the main folds while removing small details must be made with caution. Indeed, if we are interested in both sulcal and gyral patterns, it is better to simplify the entire cortical surface in a uniform manner to preserve the topology of these structures. If we are interested in a particular structure such as sulci, it is interesting in this case to simplify the surface more while preserving the topology of the sulci. So, simplification may be done without topology preservation in some subarea and with topology preservation in other ones. We introduce here the main steps performed by our method to have a rapid and efficient characterization of cortical surfaces (see Figure 3 and Algorithm 1).

### 2.2. Discrete Cortical Surface Detection

To study the human cortex folding patterns, we may either use the interface between gray matter (GM) and white matter (WM) as the cortical surface representation is reliable [13]. In recent years, there has been a considerable effort in developing methods for this purpose. A range of methods including classification-based, region-based, contour-based, and knowledge-based approaches have been proposed for MR brain image segmentation. Manual editing is highly accurate and has been one of the most employed techniques, but tedious and laborious, because boundaries are usually traced by hand. Automating this process is a challenging problem.

#### 2.2.1. Gray Matter/White Matter Interface Extraction

Recently, we have proposed an efficient fully automatic method for brain MRI volume segmentation [23]. The approach performs the segmentation using a succession of operations involving a registration step from known data, a classification step, and a segmentation step based on the level-set technique. The role of the registration and the classification is to accurately initialize the active model and to control its evolution. Recently, we have proposed a new formulation [24] for the evolution of the variational model which is expressed as

(1) $$\begin{array}{c} \frac{\partial \psi }{\partial t}=\left[\alpha_{r}F_{\text{region}}\left(I\right)+\alpha_{b}F_{\text{boundary}}\left(I\right)\right]\left|\nabla \psi \right|. \end{array}$$

*F*_boundary_ causes the evolving of the front to be more strongly attracted to image edges and is given by

(2) $$\begin{array}{c} F_{\text{boundary}}\left(I\right)=\mathrm{sign}\left(F_{\text{boundary}}\right)\cdot \frac{c+k}{1+\left|\nabla I\right|}, \end{array}$$

where

(3) $$\begin{array}{c} \mathrm{sign}\left(F_{\text{boundary}}\right)=\{\begin{array}{cc} +1, & \text{if}\;F_{\text{region}}<0,\\ -1, & \text{otherwise}. \end{array} \end{array}$$

*F*_region_ controls the evolution of the model and segments the cancer region based on the following equation:

(4) $$\begin{array}{c} F_{\text{region}}\left(I\right)=\{\begin{array}{cc} I-\left(m_{T}-\epsilon_{T}\right), & \text{if}\;I\;<\;m_{T},\\ \left(m_{T}+\epsilon_{T}\right)-I, & \text{otherwise}, \end{array} \end{array}$$

where *ϵ*_*T*_ is a constant parameter and *m*_*T*_ is the mean value of the bone cancer region. This value is calculated on the estimated region after the classification step. *ϵ*_*T*_ controls the brightness of the region to be segmented and defines a range of greyscale values that could be considered inside the expected region of interest. More technical details could be found in [23, 24].

#### 2.2.2. Decimation and Topology Preserving

Representing the surface as explicit geometry is efficient when used with the conventional computer graphics approaches for shading and viewing. Further, it greatly reduces the necessary data storage and provides a data structure that can be measured. In our case, a triangulated surface mesh was generated from the segmented white/gray matter using a standard isosurface “Marching Cubes” algorithm [25]. It has the advantage of providing an accurate three-dimensional polygonal representation that can be used for other image processing tasks. Nevertheless, the marching cubes output usually contains multiple small “useless" meshes which are physically disconnected from each other. Moreover, it produces more than the necessary number of polygons needed to represent an object accurately. The result contains an enormous number of extremely small triangles that prevent an interactive rendering of models. A high resolution mesh generated from volume data is generally very hard to work with. In order to reduce aliasing artifacts on images, we are led to apply the algorithm proposed in [26]. On the other hand, we suggest simplifying the created mesh in order to accelerate the overall mesh analyzing process. In fact, a low resolution mesh is an efficient way to convey fine surface details while maintaining a simple underlying geometry. To achieve mesh simplification, we use VTK's Quadric Decimation [27, 28], an algorithm to reduce the large number of triangles in the mesh. In Hoppe's scheme, the topology preserving operations (EdgeCollapse and EdgeSplit) are sufficient to transform the full resolution mesh into a simpler base mesh. This optimization procedure is able to provide a good approximation to the original mesh, preserves its geometry, and conserves its overall appearance (material identifiers, color values, normals, and texture coordinates). The algorithm is based on repeated edge collapses until the requested mesh reduction is achieved. Edges are placed in a priority queue based on a quadric error metric to delete the edge. So, an optimal collapse point can be computed. The process is repeated until the desired reduction level is reached or until topological constraints prevent further reduction.

### 2.3. Local Cerebral Analysis

**Figure 2:**
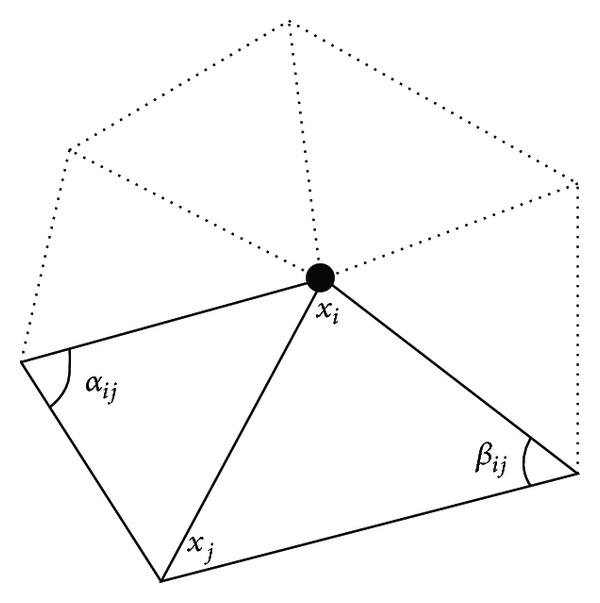
One-ring neighbors and angles opposite to an edge.

**Figure 3:**
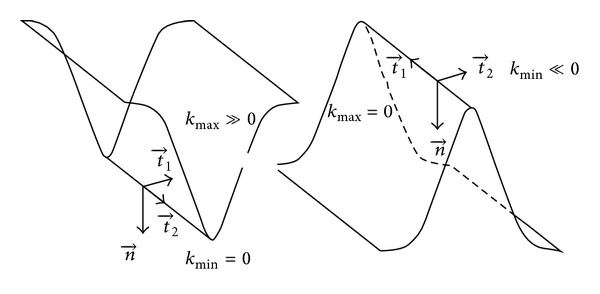
Sign of the principal curvatures relative to the surface behavior.

Different approaches can be used to study fine details of the cortical surface folding patterns. For example, depth maxima have been used to detect a concept similar to sulcal roots in [15]. On the other hand, a surface's behavior can be described by dividing the surface into distinct regions of elliptic and hyperbolic behaviors. The regions of elliptic behavior can be classified into convex and concave regions by considering the direction of the surface normal. Curvature is one of the most useful criteria for intrinsic structure description of a given surface. For example, Gaussian and mean curvatures may be used to classify the surface into meaningful structures such as valleys or ridges. Through these structures, it is possible to discriminate the surface into connected elliptical or hyperbolic regions. In this study, we suggest segmenting the discrete surface into homogeneous small regions according to different criteria which are based mainly on the local principal curvatures. We compute Gaussian and mean curvatures, and later we use them to classify the vertices into different categories. To estimate the curvature information of each vertex we have applied the approximations proposed by Meyer et al. [29]. We recall here the expression of the mean *K*_*H*_ and the Gaussian *K*_*G*_ curvature operators:

(5) $$\begin{array}{c} K_{H}\left(x_{i}\right)=\frac{1}{2}||\frac{1}{2A_{M}}\overset{}{\underset{i\in N\left(i\right)}{\sum }}\left(\text{cot}\alpha_{ij}+\text{cot}\beta_{ij}\right)\left(x_{i}-x_{j}\right)||\\ K_{G}\left(x_{i}\right)=\frac{2\Pi -\sum_{j=1}^{♯f}\Theta_{j}}{A_{M}}, \end{array}$$

where *α*_*ij*_ and *β*_*ij*_ are the two angles opposite to the edge in the two triangles sharing the edge (*x*_*i*_, *x*_*j*_) as depicted in Figure 2. Θ_*j*_ is the angle of the *j*th face at the vertex *x*_*i*_ and *♯f* denotes the number of faces around this vertex. The maximum principal curvature *k*_max⁡_ and the minimum principal curvatures *k*_min⁡_, which are related to the Gaussian and the mean curvatures, are also calculated at the vertex *x*_*i*_ as:

(6) $$\begin{array}{c} k_{\max}\left(x_{i}\right)=K_{H}\left(x_{i}\right)+\sqrt{\Delta \left(x_{i}\right)},\\ k_{\min}\left(x_{i}\right)=K_{H}\left(x_{i}\right)-\sqrt{\Delta \left(x_{i}\right)},\\ \Delta \left(x_{i}\right)=\max\left(K_{H}^{2}\left(x_{i}\right)-K_{G}^{2}\left(x_{i}\right),0\right). \end{array}$$

By this mean, each vertex should belong to a gyral or sulcal compartment based on:

(i) Its mean and Gaussian curvature values [14],
(ii) A function of the principal curvatures, *k*_max⁡_ and *k*_min⁡_, so-called Shape Index (SI) [30] which is independent of translation, rotation, and scaling. This measure is given by
  (7) $$\begin{array}{c} \text{SI}=-\frac{2}{\pi }\arctan\left(\frac{k_{\max}+k_{\min}}{k_{\max}-k_{\min}}\right). \end{array}$$

The value of the Shape Index varies in [−1, 1] where negative values represent concave surface, whereas positive values correspond to convex one. The segmentation process consists of classifying vertices according to Table 1 by encoding each surface type with a particular color. Indeed, elliptic regions on the surface must be separated from hyperbolic regions. Taking into account these criteria guarantees an efficient way to classify all vertices of the discrete surface into gyral or sulcal features.

## 3. Experimental Results

**Figure 4:**
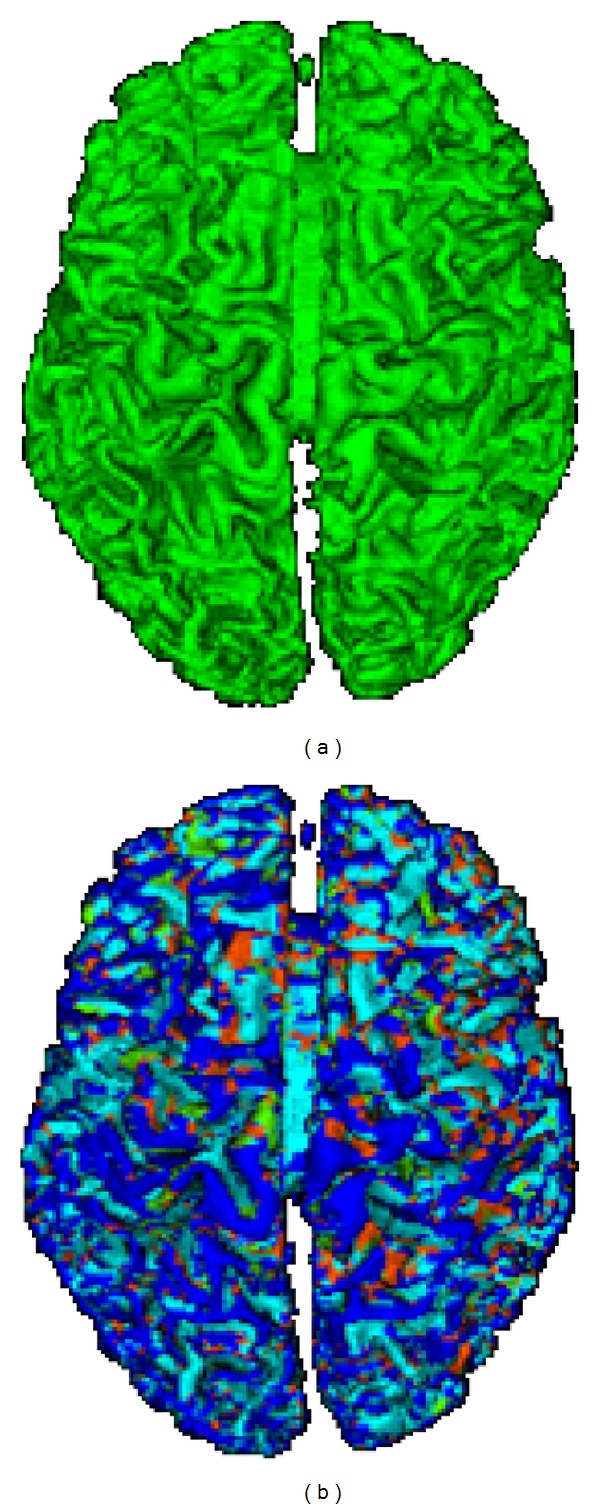
An example of local cortical surface segmentation. The blue color is associated with sulcal regions, red with minimum values of sulcal regions, green with gyral regions, and cyan with maximum values of gyral regions.

We have performed a series of experiments on brain MR images. Resulting labels are depicted in Figure 4. This figure shows the partitioning of the cortical mesh into elliptical convex regions (cyan), elliptical concave regions (red), hyperbolic convex regions (green), and hyperbolic concave regions (blue). These segmentations are presented also in a multiresolution setting (Figure 6).

**Figure 5:**
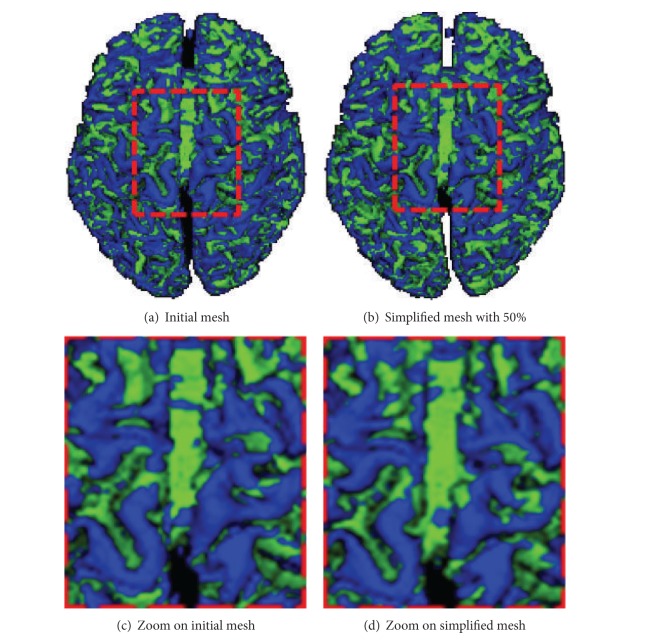
Cortex surface segmentation for two different resolutions: initial mesh and simplified mesh with 50%. This figure shows the possibility of identifying the sulcal and gyral structures even after a strong simplification.

To validate the proposed method, we limited ourselves to a qualitative assessment of results. Unfortunately, we were not able to compare our results with other works because of the lack of published papers with a complete quantitative result on the same images. We recall that we are mainly interested in investigating the ability of the method to identify the required structures in different resolutions. We present in this section some obtained results.  Figure 5 shows two results for the segmentation (classification) of the same cortex: the first result (Figure 5(a)) is associated with the initial mesh and the second one (Figure 5(b)) with the simplified mesh (with 50%). We find that sulcal and gyral regions are well-preserved and still identifiable even after a significant reduction in the initial mesh. This result justifies our choice for an intermediate step of mesh simplification.

**Figure 6:**
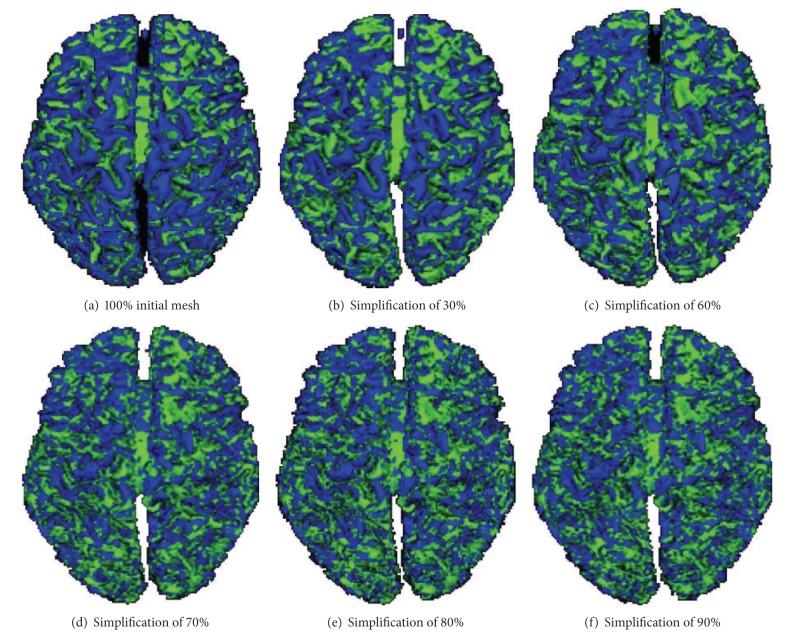
Local segmentation of cortical surface for different resolutions.

Figure 6 shows a hierarchy of the segmented cortex into gyral and sulcal structures. From this result, we see that the topology of large structures is conserved even after several simplifications of the initial mesh. It is noteworthy that this observation has been made also in [27]. It was also noted that gyral regions were better preserved than sulcal regions. Indeed, sulcal regions are thinner than gyral ones; thus, a simplifying operation will alter the topology of these small structures. From this result, we note also that the analysis (classification of the cortical surface) of low resolution has the advantage of clearly identifying the structures of interest with elliptic and hyperbolic forms. It should be noted at this point that the analysis of a higher resolution (more complex resolution) could be easily deduced from the other coarser resolutions. This reconstruction can be performed using basic inverse operators on the current mesh. Thereby, we obtain significant gain in computing time when we performed our algorithm on simplified meshes instead of initial complex ones.

The obvious difficulty in analyzing the obtained results is that there is no clear definition of what is correct? In some papers, authors rely on neurology experts to locate such sulci and gyri structures in the brain. Otherwise, the results can be interpreted based on the following idea: gyrus is located in the upper zone of the cortex (that is to say, the upper folds) and sulcus is placed in the basins of the cortex. Consequently, it will be easy to distinguish between elliptical and hyperbolic shapes. The obtained results show that it is possible to detect main features on low resolution (only 30% of the original data), whereas, if we want to do the same thing on higher resolution (100%), we cannot interpret clearly these results nor recognize the local shape of the region of interest. In fact, the classification may include small irrelevant details, which have no effect on the overall shape. On the other hand, this process is considered time-consuming since discrete surfaces are so dense. Current tests were performed on data extracted from the ICBM (International Consortium for Brain Mapping Data Base). In the future, quantitative validations will be carried out to guarantee the performance of the method. We suggest also to study other datasets showing a notable morphological variability.

## 4. Conclusion

The 3D medical image analysis, especially the cortical surface analysis problem, is both an important and difficult task. Its applications are numerous such as in neurology. The goal of this work was to develop a method for local shape segmentation based on surface curvature and multiresolution scheme. Local curvature measures properties provide an effective shape measure and could be effectively applied to discrete surface analysis. Moreover, the multiresolution way provides the neurologist with a map of the patient brain in a short time. Overall, our method shows qualitatively interesting results. Although the experimental results are satisfying, there are some future works to do. As a perspective for further work, we plan to address other issues including (1) the integration of other geometrical criteria to improve the surface characterization, (2) the consideration of the connection between all vertices having same criteria values, (3) the conduction of quantitative evaluations to demonstrate the robustness of the algorithm and (4) the comparison of our results with other methods that could benefit for further research. So, further investigations are required to extend the algorithm to a large range of meshes showing notable morphological variability.

## Figures and Tables

**Algorithm 1:**
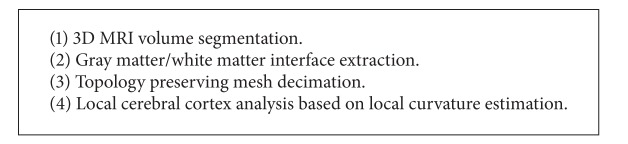
titleworktilte

**Table 1:** Possible combinations of surface types according to different descriptors.

Sign (*K*_*H*_)	−	−	+	+
Sign (*K*_*G*_)	+	−	−	+
Shape Index (SI)	[3/8, 1]	[1/8, 3/8]	[−3/8, − 1/8]	[−5/8, − 1]
Region type	Elliptic	Hyperbolic	Hyperbolic	Elliptic
Region color	Green	Cyan	Blue	Red
